# Redefining Protein Quality: Integrating Health Outcomes and Environmental Impacts in the Plant-Animal Protein Debate

**DOI:** 10.3390/foods13244128

**Published:** 2024-12-20

**Authors:** Francesco Visioli

**Affiliations:** 1 Department of Molecular Medicine, University of Padova, Viale G. Colombo 3, 35121 Padova, Italy; francesco.visioli@unipd.it; 2 IMDEA-Food, CEI UAM + CSIC, 28049 Madrid, Spain

**Keywords:** protein, sustainability, meat

## Abstract

There is an ongoing debate about the relative merits of plant-based versus animal-based protein sources in terms of human health outcomes and environmental impacts. This viewpoint article reviews and synthesizes the current evidence comparing plant and animal protein sources on measures of human health like cardiovascular disease, cancer, and mortality risk, as well as environmental factors like greenhouse gas emissions, water use, and land requirements. Overall, greater consumption of plant protein sources like legumes, nuts, seeds, and whole grains is associated with reduced risks of cardiovascular diseases, some cancers, and mortality, especially compared to red and processed meats. Crucially, these health benefits align with the dramatically lower environmental footprints of plant proteins across measures like emissions, water use, and land use. However, evidence is mixed for some health outcomes, and more research is still needed. While blanket recommendations should be avoided, the convergence of health and environmental advantages suggests future dietary guidance should emphasize shifting toward more plant-based protein sources. However, evaluations must consider specific foods rather than broad categorizations. New protein production methods like precision fermentation may also reduce environmental impacts while maintaining adequate nutrition.

## 1. Introduction

An increasing number of papers examine the effects of different animal-based and plant-based protein sources on human health outcomes and environmental impacts [1,2,3,4,5]. Most authors conclude that, overall, greater consumption of plant-based protein sources is associated with better health outcomes, particularly for cardiovascular disease risk, compared to animal-based sources [1,2,3,4,5]. Importantly, the health advantages of plant proteins align with their dramatically lower environmental footprint across measures like greenhouse gas emissions, water use, land use, and energy demands, contributing to the poorly defined sustainability goal [6]. It is indispensable to clarify some issues that are part of the One Health now-fashionable topic.

## 2. The Current Debate

It is important to underscore the traditional concept of protein quality, which is based on amino acid composition and digestibility scores like Protein Digestibility Corrected Amino Acid Score (PDCAAS) and Digestible Indispensable Amino Acid Score (DIAAS) that deem animal proteins as “high-quality” [1]. Indeed, many studies report that vegetarians have reduced risks of cardiovascular diseases such as coronary heart disease, stroke (though evidence is mixed), hypertension, type 2 diabetes, metabolic syndrome, and some cancers, like colorectal cancer [1,4,7]. However, associations are less clear for neurodegenerative diseases [4].

Nevertheless, many authors argue that these metrics fail to account for proteins’ other biological roles and environmental impacts. They suggest broadening the definition to create a new “protein quality metric” that incorporates effects on health outcomes as well as sustainability factors [8,9].

Vegetarian diets have significantly lower environmental impacts than non-vegetarian diets. Studies estimate that vegetarian patterns reduce greenhouse gas emissions by 35–87%, use 2–11% less fresh water, require 8–11% less cropland, and decrease fertilizer use by 41–46% compared to non-vegetarian diets [1,10,11,12].

Hence, should we all become vegetarian? There is no clear answer to that question. Surely, when analyzing specific animal protein sources: (a) higher intake of red and processed meat is associated with increased risks of all-cause mortality, cardiovascular diseases, type 2 diabetes, and colorectal cancer [1,13,14,15,16]. Potential mechanisms include the high saturated fat, sodium, and carcinogenic compounds formed during processing [1,13,14,15,16,17]; (b) fish intake, especially oily fish high in omega-3s, shows inverse associations with cardiovascular risks like coronary heart disease and heart failure [17,18]. However, links to diabetes, obesity, metabolic syndrome, and cancer are unclear [17,18,19]; (c) egg consumption has mixed effects, reducing some cardiovascular risks but increasing diabetes risk in several studies [20,21,22]; (d) dairy products may reduce metabolic syndrome risk but have inconsistent impacts on other cardiometabolic outcomes and cancers [23,24]. Milk type (whole vs. low-fat) influences the risk profile [23,24,25].

For plant proteins: (a) legumes, nuts/seeds, and whole grains are inversely associated with cardiovascular diseases like coronary heart disease and type 2 diabetes, with potential protective effects for some cancers like colorectal cancer [26,27]; (b) soy and soy isoflavones may reduce breast cancer risk, though evidence is limited [28,29,30].

It is worth underscoring that proper nutrition is not limited to the “animal vs. vegetal” debate. Research on human subjects has uncovered various gene–diet interactions that affect how people respond to dietary changes aimed at improving cardiovascular disease (CVD) risk factors. A wide range of dietary interventions has shown interactions with specific genetic profiles, which may help explain why individuals respond differently to the same diet. In the context of CVD, genetic variations related to lipid transport and metabolism have demonstrated significant effects. However, it is worth noting that genes involved in other biological pathways also show intriguing interactions with diet. These findings suggest that a person’s genetic makeup can influence how their body reacts to dietary changes, particularly in relation to CVD risk. This growing understanding of gene-diet interactions could potentially lead to more personalized nutritional recommendations for CVD prevention and management [31,32,33].

When directly comparing animal and plant protein sources, the limited evidence suggests shifting toward plant proteins may reduce mortality risk, especially among those with unhealthy lifestyles. However, most authors caution against overgeneralizing, as health effects vary among specific foods even within these broad categories. For example, there are some clinical conditions in the context of which adopting a vegetarian diet likely increases the risk of, e.g., vitamin B_12_ deficiency and the related reduced functioning of one-carbon metabolism, zinc and calcium deficiency, and hypoferritinemia [34]. In short, from a human health point of view, while more research is still needed, increasing consumption of plant-based protein sources appears beneficial for reducing risks of cardiovascular diseases and other chronic illnesses. This convergence of health and environmental advantages suggests future dietary guidance should emphasize shifting toward plant proteins. However, evaluations must consider the complex impacts of specific foods rather than overgeneralizing about broad categories like “animal” or “plant” proteins [35].

Limitations include the reliance on observational data, which cannot prove causality, and the heterogeneity of studies included. Additionally, many findings are based on self-reported dietary data, which has the potential for measurement error.

Environmentally, plant proteins dramatically outperform animal sources. For example, beef generates 25–26 kg of CO_2_ equivalents per kg, compared to just 0.5 kg for grains and 1.2 kg for nuts. Similarly, beef uses 8700–15,400 cubic meters of water per ton, while grains use only 1600 cubic meters [1,36,37]. This lower impact aligns with models like the Mediterranean diet “double pyramid”, depicting plant-based diets as optimal for both human and planetary health. With a view to planetary health, we must consider the overall “environmental pressure” of food production also in nutrition claims. Recent analyses have examined the main environmental factors associated with various protein sources. Generally, animal protein sources have a greater environmental impact than plant-based ones. Therefore, comparing these two broad categories is more relevant from an environmental perspective than from a strictly nutritional one.

While numerous multidisciplinary studies have thoroughly explored sustainable nutrition, the medical and health fields lack practical tools to incorporate this global health perspective into general dietary guidelines for the public. For this reason, some analyses of the main environmental impact factors of the various protein sources were carried out. It can be noted that animal protein sources generally have a greater environmental impact than plant-based ones, and, therefore, a comparison between the two macro-categories is more appropriate than strictly the nutritional field. Though several multidisciplinary studies have extensively analyzed the issue of sustainable nutrition, there is a lack of tools in the medical health field that allow us to apply this vision of global health also to broad dietary guideline advice for subjects. Finally, if we all went vegetarian and wanted to provide humanity with a high enough amount of proteins to sustain, e.g., infant growth and elderly wellbeing [38], we would need to expand arable areas and fertilizer use, with unknown consequences on the planet.

In summary, a large body of evidence indicates that plant-based protein sources like legumes, nuts, seeds, and whole grains tend to have more favorable impacts on human health, especially cardiovascular outcomes, compared to animal proteins [1]. Crucially, these health benefits converge with dramatically lower environmental footprints for plant proteins, supporting a dietary shift for both human and planetary wellbeing [4]. However, more research is still needed, and sweeping statements, particularly on the mechanisms underlying these effects and evaluating impacts of specific foods rather than broad categories, should be avoided. This chiefly applies to the as-yet poorly defined (and non-parametric) notion of sustainability, which is very difficult to quantify and is the result of many components, not limited to environmental impact [39].

In the future, the use of precision fermentation, a process that allows for the production of many complex organic molecules via microorganism reprogramming [40] to generate proteins [41,42], will likely lessen the environmental impact of protein production while maintaining adequate nutritional value. Precision fermentation uses microorganisms as “cell factories” to produce specific functional ingredients such as enzymes, flavors, and proteins. 

## 3. Conclusions

To conclude, the current hype on plant proteins as more environmentally friendly should be adequately counterbalanced by a scientifically robust analysis of their intrinsic nutritional “quality” [1,35,43,44] and—in the absence of clinical trials [45]—blanket, exaggerated pronouncements must be avoided until appropriate research and technological advancements would allow us to draw firm conclusions and provide sound recommendations.
